# CHD4 Predicts Aggressiveness in PTC Patients and Promotes Cancer Stemness and EMT in PTC Cells

**DOI:** 10.3390/ijms22020504

**Published:** 2021-01-06

**Authors:** Poyil Pratheeshkumar, Abdul K. Siraj, Sasidharan Padmaja Divya, Sandeep Kumar Parvathareddy, Khadija Alobaisi, Saif S. Al-Sobhi, Fouad Al-Dayel, Khawla S. Al-Kuraya

**Affiliations:** 1 Human Cancer Genomic Research, Research Center, King Faisal Specialist Hospital and Research Center, P.O. Box 3354, Riyadh 11211, Saudi Arabia; ppoyil@kfshrc.edu.sa (P.P.); asiraj@kfshrc.edu.sa (A.K.S.); pdivya@kfshrc.edu.sa (S.P.D.); psandeepkumar@kfshrc.edu.sa (S.K.P.); kalobaisi@kfshrc.edu.sa (K.A.); 2 Department of Surgery, King Faisal Specialist Hospital and Research Center, P.O. Box 3354, Riyadh 11211, Saudi Arabia; sobhi@kfshrc.edu.sa; 3 Department of Pathology, King Faisal Specialist Hospital and Research Center, P.O. Box 3354, Riyadh 11211, Saudi Arabia; dayelf@kfshrc.edu.sa

**Keywords:** papillary thyroid carcinoma, CHD4, disease free survival, cancer stemness, EMT

## Abstract

Chromodomain-helicase-DNA-binding protein 4 (CHD4), a core subunit of the nucleosome remodeling and deacetylation (NuRD) complex is highly expressed in several cancers. However, its role in the pathogenesis and progression of papillary thyroid carcinoma (PTC) has not been investigated. We investigated the prognostic significance of CHD4 in a large cohort of Middle Eastern PTC patients and explored the functional role of CHD4 in regulating cancer stemness and EMT in PTC cells. CHD4 overexpression was observed in 45.3% (650/1436) of PTCs, and was associated with aggressive clinico-pathological parameters and worse outcome. Functional analysis using PTC cell lines showed that forced expression of CHD4 promoted cell proliferation, spheroid growth, migration, invasion and progression of epithelial to mesenchymal transition (EMT) in PTC cells whereas its knockdown reversed the effect. Methylation of E-cadherin was associated with loss of expression in CHD4 expressing cells, while CHD4 depletion reactivated E-cadherin expression. Most importantly, knockdown of mesenchymal transcriptional factors, Snail1 or Zeb1, attenuated the spheroid growth in CHD4 expressing PTC cells, showing a potential link between EMT activation and stemness maintenance in PTC. These findings suggest that CHD4 might be a promising therapeutic target in the treatment of patients with an aggressive subtype of PTC.

## 1. Introduction

The incidence of thyroid cancer (TC) is increasing worldwide [1,2,3,4], with Saudi Arabia being no exception [5]. TC ranks as the second most common cancer among females in Saudi Arabia, accounting for 12% of all cancers [5]. Indeed, a recent study found that in the 14 most common cancer sites among male and female patients alike, incidence rates were higher among male individuals for all cancers except thyroid cancer [6]. Papillary thyroid carcinoma (PTC) is the most common histologic subtype, accounting for more than 80% of all TCs [7,8]. Among PTCs, papillary thyroid microcarcinomas (PTMC) have contributed significantly to the increased incidence of thyroid cancers in recent years [9,10]. Although there is ongoing debate about use of the term “carcinoma” for these tumors [11,12,13], it is noted that a subset of these patients may exhibit metastasis [14,15]. PTC generally displays indolent behavior and has favorable prognosis with high cure rate following initial treatment. However, recurrence is much more common, with the reported incidence ranging from 7–16% [16,17,18,19]. Therefore, identification of novel molecular targets with more selectivity could predict prognosis and helps to develop an effective method for PTC treatment.

The Chromodomain helicase DNA binding protein 4 (CHD4) is a core ATPase subunit of nucleosome remodeling and deacetylase (NuRD) complex and has a crucial role in DNA damage repair pathway [20,21]. CHD4 dysregulation has been studied in various cancer types including glioblastoma [22], breast [23], colorectal [24], lung [25,26] and rectal cancer [27]. Functionally, CHD4 has the potential to promote the growth [24,28] and migration [23,25] of several cancers. Clinically, CHD4 overexpression is associated with worse outcome in glioblastoma [22], non-small-cell lung cancer (NSCLC) [26], hepatocellular carcinoma [29], rectal cancer [27], triple negative breast cancer [23] and colorectal cancer [24]. In colorectal cancer, CHD4 activates the recruitment of DNA methyltransferases (DNMTs) to tumor suppressor gene promoters and inhibits their expression and stimulates tumorigenesis [24]. CHD4 expression is also associated with cancer stemness in hepatocellular carcinoma [29], glioblastoma [30] and endometrial cancer [31]. However, its prognostic value and functional role in PTC has not been investigated.

In the current study, we investigated the clinico-pathological and prognostic significance of CHD4 expression in a large cohort of Middle Eastern PTC patients and explored the functional role of CHD4 in PTC cell lines. Our clinical analysis results showed that CHD4 expression is correlated with aggressive clinico pathological parameters and poor disease free survival in PTC patients. In addition, our in vitro results indicated that CHD4 regulates cell growth, cancer stemness and epithelial-mesenchymal transition (EMT) in PTC cells. Thus, our study provided convincing evidence that CHD4 is a potential prognostic biomarker, and a possible target for PTC therapy.

## 2. Results

### 2.1. Patient Characteristics

Median age of the study cohort was 38 years (inter-quartile range: 29.0–50.1 years) with a female:male ratio of 3.2:1. Sixty-five percent of all cases were classical variant of PTC. Extra-thyroidal extension was noted in 43.1% of cases. Most of the cases were stage I tumors (81.4%) (Table 1).

### 2.2. CHD4 Immunoexpression and Association with Clinico-Pathological Characteristics

**Figure 1:**
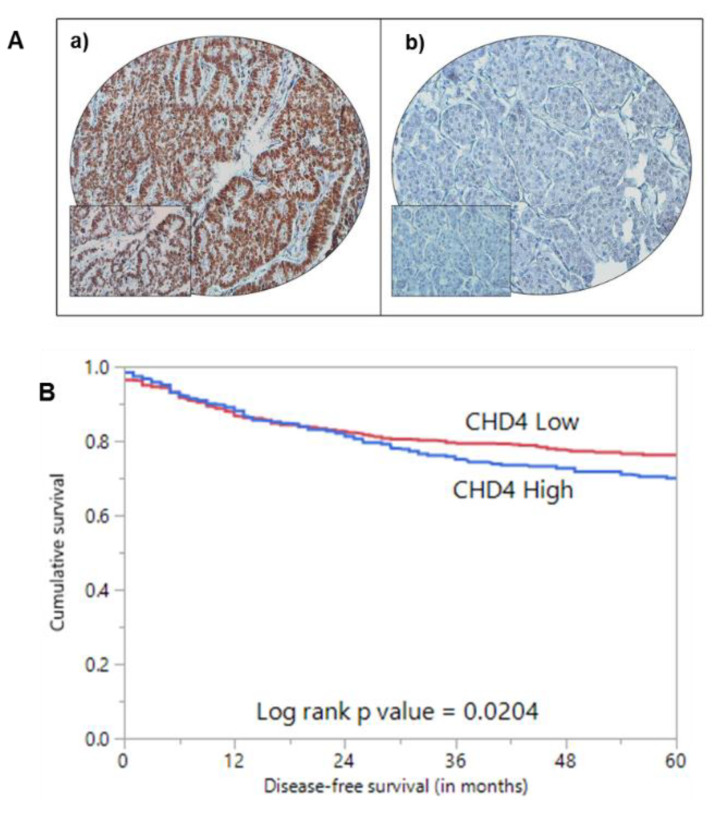
Immunohistochemical and survival analysis of CHD4 expression in Papillary Thyroid Cancer (PTC) TMA. (**A**) Representative examples of tumors showing (a) high expression and (b) low expression (right panel) of CHD4. (20×/0.70 objective on an Olympus BX 51 microscope. (Olympus America Inc., Center Valley, PA, USA) with the inset showing a 40 × 0.85 aperture magnified view of the same TMA spot). (**B**) Kaplan–Meier survival analysis for the prognostic significance of CHD4 expression in PTC. PTC patients with overexpression of CHD4 had reduced disease-free survival at 5 years compared to tumors showing low expression of CHD4 (*p* = 0.0204).

Of the 1509 PTC cases assessed, CHD4 immunoexpression was interpretable in 1436 cases. The remaining 73 cases were not interpretable due to either loss of tissue cores during processing or inadequate tumor tissue in the cores analyzed. Nuclear expression of CHD4 was considered for scoring (Figure 1A). Over-expression of CHD4 was noted in 45.3% (650/1436) of PTC cases and showed a significant association with tall cell variant (*p* < 0.0001), lymph node metastasis (*p* = 0.0085) and *BRAF* mutation (*p* < 0.0001). Importantly, over-expression of CHD4 was associated with poor 5-year disease-free survival (*p* = 0.0204) (Table 2 and Figure 1B). However, this significance was not noted in multivariate analysis after adjusting for confounding factors such as age, gender, histology, extra-thyroidal extension and stage of tumor.

### 2.3. CHD4 Promotes PTC Cell Growth In Vitro

**Figure 2:**
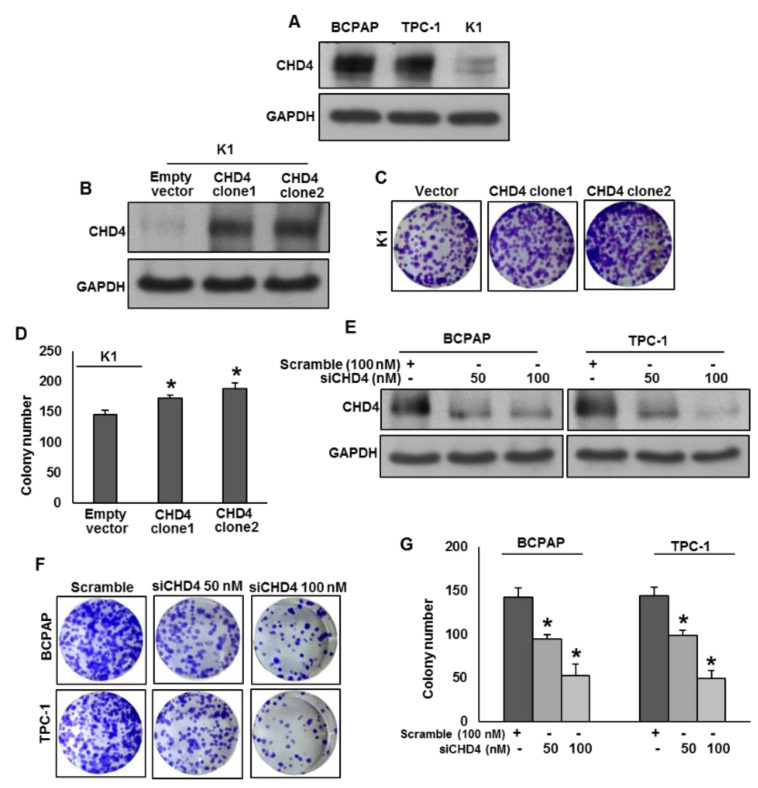
CHD4 promotes PTC cell growth in vitro. (**A**) Basal expression of CHD4 in PTC cell lines. Proteins were isolated from three PTC cell lines and immunoblotted with antibodies against CHD4 and GAPDH. (**B**) Forced expression of CHD4 in low expressing cells. K1 cells were transfected with either empty vector or *CHD4* cDNA and overexpression was confirmed by immunoblotting. (**C**,**D**) Forced expression of CHD4 increases clonogenicity. K1 cells were transfected with either empty vector or *CHD4* cDNA. After 48 h, cells were seeded at a density of 500 cells per well in 6-well plate, and grown for an additional 10 days, then stained with crystal violet and colonies were counted. (**E**) Knockdown of *CHD4* in expressing cells. PTC cells were transfected with scrambled siRNA and *CHD4* siRNA (50 and 100 nM) for 48 h. Knockdown was confirmed by siRNA. (**F**,**G**) Knockdown of CHD4 decreases clonogenicity. PTC cells were transfected with scrambled siRNA and *CHD4* siRNA (50 and 100 nM). After 48 h, cells were seeded at a density of 500 cells per well in 6-well plate, and grown for an additional 10 days, then stained with crystal violet and colonies were counted. Data presented in the bar graphs are the mean ± SD of two independent experiments. * Indicates a statistically significant difference compared to control with *p* < 0.05.

In an attempt to investigate the role of CHD4 on PTC cell growth, we first analyzed the basal expression of CHD4 in three PTC cell lines by immunoblotting (Figure 2A). We found high expression of CHD4 in BCPAP and TPC-1 cell lines, whereas K1 cells showed low or negligible expression for CHD4. Next, we overexpressed CHD4 in K1 cell line (Figure 2B) and determined the cell growth by clonogenic assay. Forced expression of CHD4 in K1 cells significantly increased the cell growth (Figure 2C,D). Conversely, Knockdown of *CHD4* in BCPAP and TPC-1 (Figure 2E) significantly decreased the cell growth (Figure 2F,G). These data demonstrate that CHD4 promotes PTC cell growth in vitro.

### 2.4. CHD4 Promotes the Self-Renewal Ability of Spheroids Generated from PTC Cells

**Figure 3:**
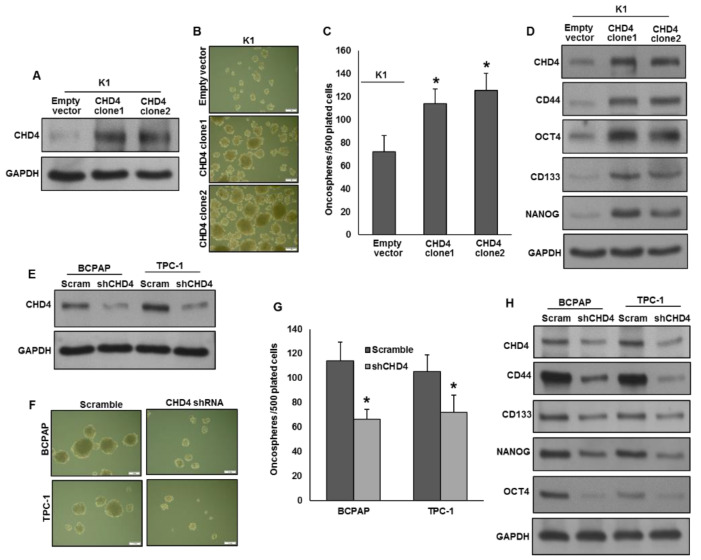
CHD4 promotes the self-renewal ability of spheroids generated from PTC cells. (**A**) Forced expression of CHD4 in low expressing cells. (**B**,**C**) Forced expression of CHD4 increases spheroid growth. K1 cells were transfected with either empty vector or *CHD4* cDNA and cells were subjected to sphere forming assay. Spheroids in the entire dish were counted. (Scale bars, 1 mm). (**D**) Forced expression of CHD4 increases stemness of spheroids as confirmed by immunoblotting using stem cell markers. K1 cells were transfected with either empty vector or *CHD4* cDNA and grown in sphere medium. Proteins were isolated from spheroids and immunoblotted with antibodies against CHD4, CD44, OCT4, CD133, NANOG and GAPDH. (**E**) Knockdown of CHD4 in expressing cells. PTC cells were transfected with *CHD4* shRNA and knockdown was confirmed by immunoblotting. (**F**,**G**) Silencing of CHD4 attenuates self-renewal ability of spheroids. PTC cells were transfected with *CHD4* shRNA and cells were subjected to sphere forming assay. Spheroids in the entire well were counted. (Scale bars, 1 mm). (**H**) Silencing of CHD4 reduces stemness of spheroids. PTC cells were transfected with *CHD4* shRNA and grown in sphere medium. Proteins were isolated from spheroids and immunoblotted with antibodies against CHD4, CD44, OCT4, CD133, NANOG and GAPDH. Data presented in the bar graphs are the mean ± SD of two independent experiments. * Indicates a statistically significant difference compared to control with *p* < 0.05.

CHD4 overexpression has been correlated with stemness and self-renewal of cancer stem cells [29,30]. To investigate the role of CHD4 in spheroid growth in PTC, we overexpressed CHD4 in low expressing cells (Figure 3A) and grown in spheroid medium. As shown in Figure 3B,C, forced expression of CHD4 significantly increased the spheroid growth. Besides, CHD4 also upregulated the expression of stem cell markers like CD44, OCT4, CD133 and NANOG as compared to empty vector transfected cells (Figure 3D). To verify the above findings, we silenced CHD4 in BCPAP and TPC-1 cells (Figure 3E) and grown in spheroid medium. As expected, knockdown of *CHD4* in these cells significantly reduced the spheroid growth (Figure 3F,G) and downregulated the stemness markers expression (Figure 3H). These data demonstrate the role of CHD4 in cancer stemness maintenance in PTC cells.

### 2.5. CHD4 Promotes Epithelial-to-Mesenchymal Transition in PTC Cells

**Figure 4:**
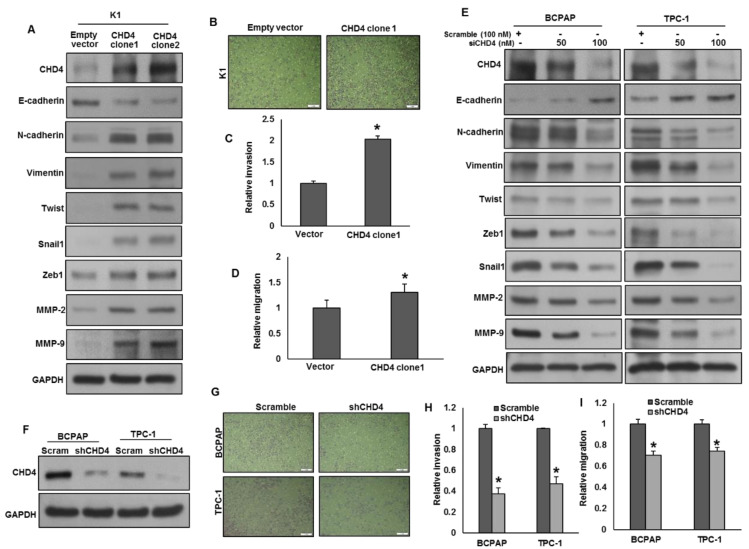
CHD4 activates epithelial-to-mesenchymal transition in PTC cells. (**A**) Forced expression of CHD4 activates EMT. K1 cells were transfected with either empty vector or CHD4 cDNA. After cell lysis, equal amounts of proteins were separated by SDS-PAGE, transferred to immobilon membrane, and immuno-blotted with antibodies against CHD4, E-cadherin, N-cadherin, Vimentin, Twist, Snail1, Zeb1, MMP-2, MMP-9 and GAPDH as indicated. (**B**,**C**) Forced expression of CHD4 increases invasion. K1 cells were transfected with either empty vector or *CHD4* cDNA. Cells were seeded into the upper compartment of invasion chambers. The bottom chambers were filled with RPMI media. After 24 h incubation, invaded cells were fixed, stained and quantified. (Scale bars, 1 mm). (**D**) Forced expression of CHD4 increases migration. K1 cells were transfected with either empty vector or *CHD4* cDNA. Cells were seeded into the upper compartment of migration chambers. The bottom chambers were filled with RPMI media. After 24 h incubation, migrated cells were fixed, stained and quantified. (**E**) Silencing of CHD4 down-regulates the expression of EMT markers in PTC cells. PTC cells were transfected with scrambled siRNA and *CHD4* siRNA (50 and 100 nM). After 48 h, cells were lysed and proteins were immunoblotted with antibodies against CHD4, E-cadherin, N-cadherin, Vimentin, Twist, Snail1, Zeb1, MMP-2, MMP-9 and GAPDH. (**F**) Knockdown of CHD4 in expressing cells. (**G**,**H**) Knockdown of CHD4 decreases invasive ability of PTC cells. PTC cells were transfected with *CHD4* shRNA and cells were subjected to invasion assay. (Scale bars, 1 mm). (**I**) Knockdown of CHD4 decreases migratory ability of PTC cells. PTC cells were transfected with *CHD4* shRNA and cells were subjected to migration assay. Data presented in the bar graphs are the mean ± SD of two independent experiments. * Indicates a statistically significant difference compared to control with *p* < 0.05.

A recent study showed that CHD4 activates EMT and induces metastatic potential in triple-negative breast cancer cells [23]. Therefore, we sought to determine whether overexpression of CHD4 has any effect on the activation of EMT in PTC cell lines. Forced expression of CHD4 in K1 cells (low expressing cells) increased the expressions of CHD4, N-cadherin, vimentin, Twist, Snail1, Zeb1, MMP-2, and MMP-9 with an associated downregulation of E-cadherin expression in K1 cells (Figure 4A). Furthermore, forced expression of CHD4 in K1 cells also increased invasion (Figure 4B,C) and migration (Figure 4D). Conversely, knockdown of CHD4 in BCPAP and TPC-1 downregulated the expressions of CHD4, N-cadherin, vimentin, Twist, Snail1, Zeb1, MMP-2, and MMP-9 with a concomitant upregulation of E-cadherin expression (Figure 4E). As expected, silencing of CHD4 (Figure 4F) also decreased invasion (Figure 4G,H) and migration (Figure 4I) of these cells. These findings indicate that CHD4 activates EMT and induces metastatic potential in PTC cells.

### 2.6. DNA Methylation-Induced E-Cadherin Silencing Is Correlated with CHD4 Expression

**Figure 5:**
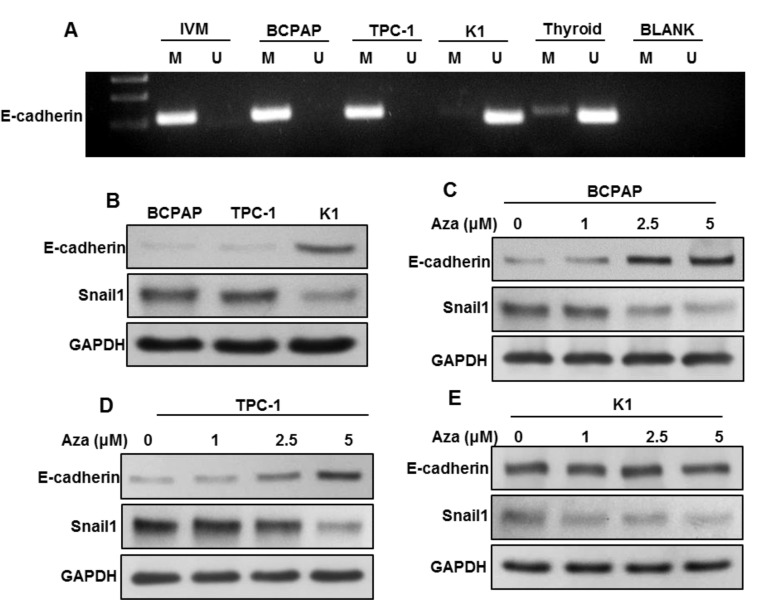
Methylation status of PTC cell lines by MSP for the *E-cadherin* gene. (**A**) Methylation status of three PTC cancer cell lines assessed by MSP for the *E-cadherin* gene. MSP analyses of both methylated (M) and unmethylated (U) reactions were amplified from bisulfite-treated DNA and run in a 2% agarose gel. (**B**) E-cadherin and Snail1 protein levels were determined by Western blotting in PTC cell lines. PTC cells were lysed and equal amounts of proteins were separated by SDS-PAGE, transferred to Immobilon membrane, and immunoblotted with antibodies against E-cadherin, Snail1 and GAPDH. (**C**,**D**) The demethylation restore E-cadherin expression in BCPAP and TPC-1 cells. PTC cells were treated with different doses (1, 2.5 and 5 μM) of 5-aza-2′deoxycytidine for 72 h and cells were lysed. Equal amounts of proteins were separated by SDS-PAGE, transferred to Immobilon membrane, and immunoblotted with antibodies against E-cadherin, Snail1 and GAPDH. (**E**) There was no change in the expression of E-cadherin and Snail1 in K1 cells after treatment with 5-aza-2′-deoxycytidine.

CHD4 has been shown to maintain the epigenetic suppression of multiple tumor suppressor genes including *E-cadherin* [24]. In an attempt to dissect the mechanism by which CHD4 induces EMT in PTC, we first determined the methylation status of *E-cadherin* promoter region in PTC cell lines (BCPAP, TPC-1 and K1) using Methylation-specific Polymerase chain reaction (MSP-PCR). Our results showed complete methylation of *E-cadherin* in CHD4 expressing cells (BCPAP and TPC-1), whereas CHD4 low expressing cells (K1) was unmethylated for *E-cadherin* gene promoter (Figure 5A). To test whether methylation of PTC cell lines cause the loss of protein expression of E-cadherin, lysates from PTC cell lines were separated on SDS-PAGE and immunoblotted with E-cadherin antibody. In concordance with MSP data, BCPAP and TPC-1 cell lines that found to be completely methylated for *E-cadherin* promoter gene showed diminished expression of E-cadherin protein, whereas K1 cells that was found to be unmethylated had appreciable expression of E-cadherin protein as detected by immunoblotting (Figure 5B). Contrarily, we found an abundant expression of Snail1, a mesenchymal transcription factor in BCPAP and TPC-1 cell lines and low expression in K1 cells (Figure 5B). These data indicate that the functionality of the *E-cadherin* gene depends on the methylation status of PTC cells. In an attempt to restore methylated *E-cadherin*, BCPAP and TPC-1 cells were treated with different doses (1, 2.5 and 5 μM) of 5-aza-2′-deoxycytidine, a demethylating agent for 3 days. As shown in Figure 5C, 5-aza-2′-deoxycytidine treatment restored E-cadherin protein expression in BCPAP (Figure 5C) and TPC-1 cells (Figure 5D) as detected by immunoblotting. We also found a decreased expression of Snail1 in BCPAP (Figure 5C) and TPC-1 cells (Figure 5D) after 5-aza-2′-deoxycytidine treatment. However, there was no change in the expression of E-cadherin and Snail1 in K1 cells after treatment with 5-aza-2′-deoxycytidine (Figure 5E). These data suggest that loss of E-cadherin protein is a result of *E-cadherin* methylation.

### 2.7. Silencing of Snail1 and Zeb1 Suppresses Self-Renewal Ability of Spheroids Generated from PTC Cells

**Figure 6:**
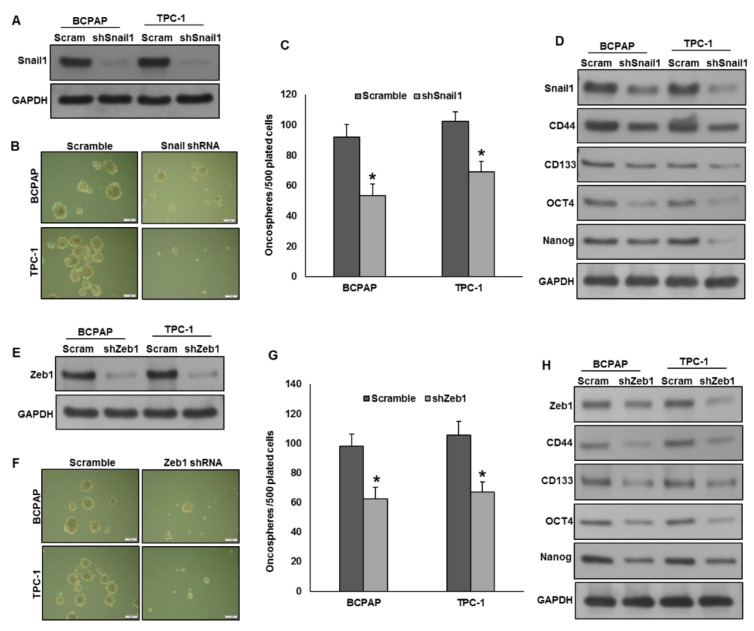
Silencing of Snail1 and Zeb1 suppresses self-renewal ability of spheroids generated from PTC cells. (**A**) Knockdown of Snail1. PTC cells were transfected with *Snail1* shRNA and knockdown was confirmed by immunoblotting. (**B**,**C**) Silencing of Snail1 suppresses spheroid growth. PTC cells were transfected with *Snail1* shRNA and cells were subjected to sphere forming assay. Spheroids in the entire dish was counted. (Scale bars, 1 mm). (**D**) Silencing of Snail1 reduces stemness of spheroids. PTC cells were transfected with *Snail1* shRNA and grown in sphere medium. Proteins were isolated from spheroids and immunoblotted with antibodies against Snail1, CD44, CD133, OCT4, NANOG and GAPDH. (**E**) Knockdown of Zeb1. PTC cells were transfected with *Zeb1* shRNA and knockdown was confirmed by immunoblotting. (**F**,**G**) Silencing of Zeb1 suppresses spheroid growth. PTC cells were transfected with *Zeb1* shRNA and cells were subjected to sphere forming assay. Spheroids in the entire dish was counted. (Scale bars, 1 mm). (**H**) Silencing of Zeb1 reduces stemness of spheroids. PTC cells were transfected with *Zeb1* shRNA and grown in sphere medium. Proteins were isolated from spheroids and immunoblotted with antibodies against Zeb1, CD44, CD133, OCT4, NANOG and GAPDH. Data presented in the bar graphs are the mean ± SD of two independent experiments. * Indicates a statistically significant difference compared to control with *p* < 0.05.

Mesenchymal transcriptional factors, Snail1 and Zeb1 are shown to downregulate E-cadherin expression in epithelial cells [32,33]. We showed that CHD4 promotes stemness and EMT progression in PTC cells. Next, we sought to determine whether EMT has any effect on spheroid growth in PTC cells. Therefore, we knockdown Snail1 and Zeb1 in CHD4 expressing cells and grown in spheroid medium. Knockdown of Snail1 (Figure 6A) significantly decreased the spheroid growth (Figure 6B,C) as well as stemness properties as indicated by downregulation of stem cell markers (Figure 6D). Similarly, knockdown of Zeb1 (Figure 6E) also decreased the self-renewal ability of spheroids (Figure 6F,G) and stemness characteristics (Figure 6H) in these cells. These findings provide the link between EMT progression and stemness maintenance in PTC cells and CHD4 might have a key role in this process.

## 3. Discussion

In the present study, we used a large cohort of PTC patient samples to address the clinico-pathological and prognostic significance of CHD4. We demonstrated overexpression of CHD4 in 45.3% (650/1436) of PTC cases. Previous studies from other organ sites have reported a similar frequency, ranging from 35%–59% [23,29,34]. Interestingly the PTC patients with CHD4 overexpression showed aggressive clinical behavior, such as tall cell variant and lymph node metastasis and poor outcome. Several previous studies have reported the association of CHD4 overexpression with aggressive markers and worse outcome in other cancers [22,23,24,26,27,29]. These findings suggest that CHD4 might be a potential prognostic marker in Middle Eastern PTC.

CHD4 has been reported to exert pro-oncogenic activity by promoting tumor progression in several types of cancers [24,25,26,28,35,36]. Our results indicate that forced expression of CHD4 significantly increased the cell growth in CHD4 low expressing cells. Conversely, knockdown of CHD4 markedly reduced the cell growth of CHD4 expressing PTC cells. These data confirm the pro-oncogenic role of CHD4 in PTC cells. Furthermore, we revealed that forced expression of CHD4 increased stemness expression of spheroids. On the contrary, CHD4 knockdown markedly decreased the spheroid growth and stemness markers expressions, verifying the role of CHD4 in maintaining cancer stemness in PTC cells. These findings are in concordance with the previous reports in hepatocellular carcinoma [29], glioblastoma [30] and endometrial cancer [31].

EMT plays an important role in tumorigenesis where the EMT program augments metastasis, tumor stemness and resistance to chemotherapy [37,38,39]. TGF-β is a known inducer for EMT, which plays a dual role in human cancers, act as both tumor promoter and suppressor depending on cellular context [40,41]. In endometrial cancer, *CHD4* mutation has been shown to promote tumorigenesis via TGF-β signaling pathway [31]. We have analyzed for *CHD4* pathogenic mutations from our previously analyzed exome data addressing 245 PTC cases and found *CHD4* is rarely mutated in PTC showing only one mutated case (0.41%, 1/245) (data not shown). We showed that forced expression of CHD4 activated EMT progression as well as increased invasion and migration in PTC cells. Luo et al. [23] also demonstrated similar findings in triple-negative breast cancer cells. It has been showed that CHD4 maintains the epigenetic silencing of tumor suppressor genes including *E-cadherin* [24,42]. Therefore, methylation status of the promoter region of *E-cadherin* gene was evaluated in three PTC cell lines. MSP analysis revealed that CHD4 expressing cell lines were found to be completely methylated, whereas CHD4 low expressing cell line was unmethylated for the *E-cadherin* gene. As expected, methylation decreased E-cadherin expression and functionality. These findings explore the mechanism by which CHD4 regulates EMT in PTC cell lines. To address the possible association between the EMT and stemness, we knockdown mesenchymal transcriptional factors, Snail1 or Zeb1 in CHD4 expressing cells and grown in spheroid growth medium. Interestingly, knockdown of mesenchymal transcriptional factors, Snail1 or Zeb1, attenuated the spheroid growth and stemness properties in CHD4 expressing PTC cells, showing a potential link between EMT activation and stemness maintenance in PTC.

## 4. Materials and Methods

### 4.1. Patient Selection

One thousand five hundred and nine (1509) patients with PTC diagnosed between 1988 and 2018 were selected from King Faisal Specialist Hospital and Research Centre. However, immunohistochemical analysis could be performed in only 1436 cases and hence were included for further analysis. Detailed clinico-pathological data were noted from case records and have been summarized in Table 1. Institutional Review Board approved the acquisition of all archival tissue samples. Since only archival tissue samples were utilized for this study, a waiver of consent was obtained from Research Advisory Council under project RAC#2170022 on 13 Nov 2017.

### 4.2. Tissue Microarray (TMA) Construction and Immunohistochemistry

Tissue microarray (TMA) format was used to analyze protein expression in all samples. Construction of TMA was performed as described previously [43]. A modified semiautomatic robotic precision instrument (Beecher Instruments, Woodland, WI, USA) was used to punch a tissue cylinder of 0.6 mm diameter from representative tumor regions of the donor block. The tissue cylinders were then transferred to the recipient block. Two cores of tumor were arrayed from each case.

Processing and staining of tissue microarray slides was performed manually, as described earlier [44]. Antigen retrieval was performed using Dako (Dako Denmark A/S, Glostrup, Denmark) Target Retrieval Solution at pH 9.0 (Catalog number S2367). Slides were placed in Pascal pressure cooker for 8 min at 120℃. Primary antibody against CHD4 (ab72418, Abcam, Cambridge, UK) at a dilution of 1:8000 (pH 9.0) was used for staining the slides. The Dako Envision Plus System kit was used as the secondary detection system with 3, 30-diaminobenzidine as chromogen. The slides were then counterstained with hematoxylin, dehydrated in alcohol, cleared using xylene and mounted. Staining of control slides with omission of primary antibody served as negative controls. Breast cancer tissues were used as positive controls.

Scoring for CHD4 immunoreactivity was performed as described previously [24]. Briefly, the intensity of positively stained cells was 0 if no staining was detected, 1 for weak staining, 2 for moderate staining and 3 for strong staining. Proportion of positively stained cells was scored as 0 if no staining was detected, 1 if 1%–25% of tumor cells were positively stained, 2 if 26%–50% of tumor cells were positively stained, 3 if 51%–75% of tumor cells were positively stained and 4 if 76%–100% of tumor cells were positively stained. The final immunoreactivity scores were calculated by multiplying the intensity and proportion scores. The final score ranged from 0–12. Score of 0–3 was considered as “low expression” and 4–12 was considered as “over expression”.

### 4.3. Bisulfite Modification and Methylation-Specific PCR

Genomic DNA isolated from PTC cells was subjected to bisulfite modification using an EZ DNA Methylation Kit (Zymo Research, Orange, CA, USA) according to manufacturer’s instructions [45]. Briefly, 200 ng of DNA was treated with CT conversion reagent and incubated in a thermal cycler at 98 °C for 8 min followed by incubation at 64 °C for three and a half hours. The reaction was then stored at 4 °C until used for the next step. The bisulfite converted DNA was subjected to methylation-specific PCR (MSP) using primers specific for CpG islands in the *E-cadherin* promoter: forward primer, 5′-TTAGGTTAGAGGGTTATCGCGT-3′ and reverse primer, 5′-TAACTAAAAATTCACCTACCGAC-3′. For unmethylated DNA: forward primer, 5′-TAATTTTAGGTTAGAGGGTTATTGT-3′ and reverse primer, 5′-CACAACCAATCA ACAACACA-3′. MSP products of *E-cadherin* methylation and unmethylation were analyzed on 2% agarose gel after staining with ethidium bromide and visualized under UV-light.

### 4.4. Cell Culture

BCPAP cell line was purchased from from Deutsche Sammlung von Mikroorganismen und Zellkulturen (DSMZ, Braunschweig, Germany). Cell line, TPC-1 was generously gifted by Dr. Bryan McIver (Department of Endocrinology, Mayo Clinic, Rochester, MN, USA). K1 cell line was obtained from American Type Culture Collection (ATCC, Manassas, VA, USA.). K1 cell line was originally isolated from PTC male patient and has been classified as Misidentified Cell Lines by International Cell Line Authentication Committee (ICLAC-00218). All the cell lines were maintained in CO_2_ (5%) incubator with humidified atmosphere at 37 °C. Using short tandem repeats (STR) DNA profiling, cell lines were authenticated in-house and the results were in concordance with published data [46,47]. In vitro studies were conducted using serum reduced RPMI media (5% FBS).

### 4.5. Reagents and Antibodies

Antibodies against CHD4 (ab72418), N-cadherin (ab19348), Vimentin (ab92547) and Twist (ab175430) were purchased from Abcam Inc. (Cambridge, MA, USA). Antibodies against E-cadherin (3195), OCT4 (75463), Nanog (4903), CD44 (3570), CD133 (64326), MMP-2 (4022) and MMP-9 (2270) were purchased from Cell Signaling Technology (Danvers, MA, USA). Snail1 antibody (MA5-14801) was purchased from Thermo Fisher Scientific (Rockford, IL, USA). Zeb1 antibody (NBP1-05987) was purchased from Novus Biologicals (Minneapolis, MN, USA). GAPDH (sc-25778) antibody was purchased from Santa Cruz Biotechnology, Inc. (Santa Cruz, CA, USA).

### 4.6. Clonogenic Assay

The PTC cell lines were seeded at a density of 500 cells per well in 6-well plate. After attachment, fresh growth medium was added and cells were allowed to grow for 8–10 days. Cell colonies were fixed with formaldehyde (4%) and stained with crystal violet (2% in 10% methanol). The number of colonies in each well were counted and photographed.

### 4.7. Gene Knockdown Using siRNA

PTC cell lines were transiently transfected with *CHD4* siRNA and scrambled control siRNA (OriGene, Rockville, MD, USA) using Lipofectamine 2000 (Invitrogen, Carlsbad, CA, USA). Six hours post transfection, lipid-siRNA complex was replaced with fresh growth medium. After 48 h of transfection, knockdown efficiency was confirmed by immunoblotting.

### 4.8. Plasmid and Transfection

Plasmid DNA encoding human *CHD4* and shRNA’s targeting human *CHD4*, *Snail1* and *Zeb1* were purchased from Origene (Rockville, MD, USA). The overexpression of *CHD4* and knockdown of *CHD4, Snail1* and *Zeb1* in PTC cells were conducted using Lipofectamine™2000 (Invitrogen, Carlsbad, CA, USA). Briefly, PTC cells (1 × 10^5^) were plated in 6-well culture plates and cells were transfected with 4 μg plasmid for 48 h. The overexpression of CHD4 and knockdown of CHD4, Snail1 and Zeb1 protein production were confirmed by immunoblotting.

### 4.9. Trans-Well Cell Invasion and Migration Assays

The trans-well invasion and migration assays were conducted as described previously [47]. Briefly, cells after transfection, 100 μL of cells (5 × 10^4^) were inoculated into the upper chamber of trans-well inserts either coated with matrigel matrix (BD Biosciences) (for invasion assay) or uncoated (for migration assay). The lower chamber was added with 500 μL of complete medium. Twenty-four hours post incubation, the non-invalided cells were removed. The cells that had invaded or migrated through the membrane were fixed and stained using Diff-Quick stain set (Fisher Scientific, Pittsburg, PA, USA). Finally, invaded or migrated cells were photographed and counted under a microscope.

### 4.10. Sphere Forming Assay

PTC cells (500/well) were plated on Corning 24-well ultra-low attachment plates (Sigma-Aldrich) grown in serum free DMEM-F12 (ATCC) supplemented with B27 (Thermo Fisher), 20 ng/mL epidermal growth factor (Sigma-Aldrich), 0.4% bovine serum albumin (Sigma-Aldrich) and 4 μg/mL insulin (Sigma-Aldrich). Fresh medium was supplemented every 2 days. The spheroids were counted and photographed at day 14. For secondary spheroid formation, the primary spheroids were dissociated into single cells, and cultured on 24-well ultra-low attachment plates using spheroid culture medium for another 10 days.

### 4.11. Statistical Analysis

Associations between clinico-pathological variables and CHD4 protein expression was analyzed performed using contingency table analysis and chi-square tests. Kaplan–Meier method was used to generate disease-free survival curves and Mantel–Cox log-rank test was used to evaluate significance. A *p*-value of < 0.05 was defined as limit of significance for all statistical analyses. Two-sided tests were performed for all calculations. Statistical analyses was performed using the JMP14.0 (SAS Institute, Inc., Cary, NC, USA) software package.

For all in vitro studies, data presented in the bar graphs are the mean ± SD of duplicates in an independent experiment, which was repeated at least twice with the same results. Student *t* test (two-tailed) was performed for statistical significance with a *p* < 0.05 used as the cut-off.

## 5. Conclusions

In summary, we demonstrated overexpression of CHD4 in Middle Eastern PTC patient samples, which was further correlated with aggressive clinico-pathological parameters and poor outcome. The data clearly demonstrate that CHD4 plays an important role in PTC pathogenesis. We showed the role of CHD4 in tumor progression, metastasis and stemness in PTC cells. Furthermore, our study also dissected the link between EMT progression and stemness maintenance in CHD4 expressing PTC cells. These findings suggest that CHD4 may be a promising therapeutic target in the treatment of patients with an aggressive subtype of PTC.

## Figures and Tables

**Table 1:** Clinicopathological variables for the patient cohort (*n* = 1436).

Clinico-Pathological Variables	*n* (%)
**Age**	
Median	38.0
Range (IQR) ^	29.0–50.1
**Gender**	
Female	1090 (75.9)
Male	346 (24.1)
**Histopathology**	
Classical Variant	936 (65.2)
Follicular Variant	256 (17.8)
Tall Cell Variant	131 (9.1)
Hobnail Variant	1 (0.1)
Others	112 (7.8)
**Extra Thyroidal Extension**	
Present	619 (43.1)
Absent	817 (56.9)
**Tumor size**	
≤1 cm	137 (9.5)
>1 cm	1246 (86.8)
Unknown	53 (3.7)
**pT**	
T1	396 (27.6)
T2	289 (20.1)
T3	588 (40.9)
T4	110 (7.7)
Unknown	53 (3.7)
**pN**	
N0	582 (40.6)
N1	710 (49.4)
Nx	144 (10.0)
**pM**	
M0	1291 (89.9)
M1	55 (3.8)
Mx	90 (6.3)
**Stage**	
I	1169 (81.4)
II	156 (10.9)
III	20 (1.4)
IVA	19 (1.3)
IVB	28 (1.9)
Unknown	44 (3.1)

Abbreviations—^ Inter quartile range.

**Table 2:** Clinico-pathological associations of CHD4 protein expression in Papillary Thyroid Carcinoma.

	Total	CHD4 Over-Expression	CHD4 Low Expression	*p* Value
	*n* (%)	*n* (%)	*n* (%)	
**No. of patients**	1436	650 (45.3)	786 (54.7)	
**Age (Yrs)**				
<55	1169 (81.4)	524 (44.8)	645 (55.2)	0.4837
≥55	267 (18.6)	126 (47.2)	141 (52.8)	
**Sex**				
Female	1090 (75.9)	476 (43.7)	614 (56.3)	**0.0314**
Male	346 (24.1)	174 (50.3)	172 (49.7)	
**Histology Type**				
Classical Variant	936 (65.2)	437 (46.7)	499 (53.3)	**<0.0001**
Follicular Variant	256 (17.8)	88 (34.4)	168 (65.6)	
Tall-Cell Variant	131 (9.1)	78 (59.5)	53 (40.5)	
Other variants	113 (7.9)	47 (41.6)	66 (58.4)	
**Extrathyroidal extension**				
Present	619 (43.1)	297 (48.0)	322 (52.0)	0.0720
Absent	817 (56.9)	353 (43.2)	464 (56.8)	
**Tumor size**				
≤1 cm	137 (9.9)	57 (41.6)	80 (58.4)	0.3451
>1 cm	1246 (90.1)	571 (45.8)	675 (54.2)	
**pT**				
pT1	396 (28.6)	175 (44.2)	221 (55.8)	0.2923
pT2	289 (20.9)	122 (42.2)	167 (57.8)	
pT3	588 (42.5)	284 (48.3)	304 (51.7)	
pT4	110 (8.0)	47 (42.7)	63 (57.3)	
**pN**				
pN0	582 (45.1)	237 (40.7)	345 (59.3)	**0.0085**
pN1	710 (54.9)	341 (48.0)	369 (52.0)	
**pM**				
pM0	1291 (95.9)	575 (44.5)	716 (55.5)	0.3498
pM1	55 (4.1)	21 (38.2)	34 (61.8)	
**Stage**				
I	1169 (84.0)	524 (44.8)	645 (55.2)	0.4698
II	156 (11.2)	74 (47.4)	82 (52.6)	
III	20 (1.4)	11 (55.0)	9 (45.0)	
IVA	19 (1.4)	10 (52.6)	9 (47.4)	
IVB	28 (2.0)	9 (32.1)	19 (67.9)	
***BRAF*** **mutation**				
Positive	704 (56.5)	407 (57.8)	297 (42.2)	**<0.0001**
Negative	543 (43.5)	167 (30.8)	376 (69.2)	
**Disease Free Survival**				
5 years		70.0	76.2	**0.0204**

Of the 1509 PTC cases assessed, CHD4 immunoexpression was interpretable in 1436 cases.

## Data Availability

The data presented in this study are available on request from the corresponding author.
